# Impact of a Mental Health Diagnosis on Emergency Department Adherence to Sepsis Care Guidelines

**DOI:** 10.31486/toj.21.0091

**Published:** 2022

**Authors:** Kathleen A. Crapanzano, Rebecca Hammarlund, Mandi Musso, Leah Dlugolecki, Lauren Luce, Ross Divers, LuAnn Barnett, Claude D’Antonio

**Affiliations:** ^1^ Department of Psychiatry, Louisiana State University Health Sciences Center, Baton Rouge, LA; ^2^ Division of Academic Affairs, Our Lady of the Lake Regional Medical Center, Baton Rouge, LA; ^3^ Department of Emergency Medicine, Louisiana State University Health Sciences Center, Baton Rouge, LA; ^4^ Department of Psychology, Louisiana State University, Baton Rouge, LA

**Keywords:** *Mental disorders*, *sepsis*, *social stigma*

## Abstract

**Background:** Previous work has found that clinical care for a variety of health conditions varies depending upon the mental health status of the patient. Sepsis, a condition with an algorithm-driven care plan, has not yet been investigated. This study sought to determine if disparities in care exist for people with mental illness and suspected sepsis.

**Methods:** We conducted a retrospective medical records review of patients presenting to the emergency department with a clinical suspicion of sepsis from June 1, 2017, to January 31, 2018. Extracted data included clinical care decisions consistent with the Severe Sepsis and Septic Shock Early Management Bundle (SEP-1) national guidelines and information from the problem list and encounter notes about the presence of mental illness.

**Results:** Seven hundred ninety-eight patient encounters were included in the study. Sixty-eight percent of these encounters had care that met the 3-hour SEP-1 bundle guidelines. The presence of a psychiatric diagnosis was not significantly related to failure of SEP-1 criteria, χ^2^(1)=1.01, *P*=0.315.

**Conclusion:** This study showed no differences in clinical decision-making for patients with sepsis and a psychiatric diagnosis of mental illness. The presence of objective guidelines may have lessened the potential role of biases among clinicians toward patients with mental illness.

## INTRODUCTION

Compared to the general population, individuals with severe mental illness have shortened lifespans.^1-3^ Further, individuals with mental illness frequently encounter stigma in the health care setting that may impact the care provided and ultimately patient outcomes.^4,5^ For example, studies indicate that individuals with severe mental illness have higher mortality rates after infection compared to individuals without mental illness.^3,6-9^ Previous work has posited that physicians may misattribute symptoms of medical disorders to mental illness and be reluctant to assess or treat medical conditions, an attitude that could contribute to these worsened outcomes.^10^

One of the most significant complications of infection is sepsis, defined as “a life-threatening organ dysfunction caused by a dysregulated host response to infection.”^11,12^ In-hospital mortality of severe sepsis is as high as 30%.^13,14^ Research indicates that early treatment of sepsis with rapid administration of broad-spectrum antibiotics can improve in-hospital mortality.^15,16^

The Centers for Medicare and Medicaid Services (CMS) began measuring hospital responses to the treatment of septic patients using a bundle of care called the Severe Sepsis and Septic Shock Early Management Bundle (SEP-1) on October 1, 2015. The SEP-1 guidelines recommend that patients suspected of sepsis have lactate and blood cultures collected and initial broad-spectrum antibiotics started within 3 hours of sepsis indications being noted by providers.^17^ Additional treatment recommendations are made for 6 hours after presentation and for patients with septic shock. In 2018, CMS began publicly reporting hospital metrics regarding assessment and treatment of severe sepsis and septic shock within 3 and 6 hours.^17^

A paucity of data is available regarding the treatment of persons presenting to the emergency department (ED) for infection with comorbid mental illness. The purpose of this study was to compare one hospital's compliance with the 3-hour SEP-1 guidelines in patients with and without mental illness. We hypothesized that physicians would be less compliant with SEP-1 guidelines for patients with mental illness.

## METHODS

This retrospective medical records review was approved by the Institutional Review Board at Louisiana State University Health Sciences Center–New Orleans.

### Encounter List

Using the Slicer Dicer search tool available in the Epic electronic medical record (Epic Systems Corporation), an encounter list was generated for the 3 Louisiana hospitals of a large health care system. Using the ED encounters view, 1,633 encounters with patients 18 to 90 years old with an ED diagnosis (primary or other) of sepsis or systemic inflammatory response syndrome between June 1, 2017, and January 31, 2018, were identified. This time period was chosen because it was representative of care before the commencement of a quality improvement project to address adherence to SEP-1 criteria.

Of the 1,633 encounters, 113 encounters involved repeat patients. The first encounter for each patient was retained, and subsequent encounters were deleted from the encounter list. A further 487 encounters were deleted from the encounter list for the following reasons: patient had dementia or a significant cognitive disability resulting in baseline nonverbal status before the encounter (n=201); patient was a transfer from an outside facility (n=188); patient died, was admitted, or left against medical advice before 3 hours (n=53); patient was on hospice or palliative care (n=36); sepsis was not mentioned in the encounter note (n=5); and problems were identified with the medical record (n=4). Thus, 1,033 encounters were examined for adherence to SEP-1 3-hour bundle criteria.

### Time 0 Events

Given that SEP-1 3-hour bundle criteria are specific to cases of severe sepsis, we defined Time 0 events for this study as any 1 of 8 of the organ dysfunction variables defined in the SEP-1 guidelines:^18^ systolic blood pressure <90 mm Hg, mean arterial pressure <70 mm Hg, creatinine >2.0 mg/dL (reference range, 0.57-1.25 mg/dL), bilirubin >2.0 mg/dL (reference range, 0.2-1.2 mg/dL), platelets <100 K/μL (reference range, 150-375 K/μL), international normalized ratio >1.5 or partial thromboplastin time >60 sec (reference range, 21-30 sec), altered mental status, or lactate >2.0 mmol/L (reference range, 0.5-2.2 mmol/L). When multiple organ dysfunction variables were present, the time of the earliest one was considered Time 0.

### SEP-1 3-Hour Bundle Criteria

Once a Time 0 event was identified, the preceding 3 hours of the care timeline were searched for the presence of a lactate test, a blood culture draw, and administration of an antibiotic after the blood draw. If each component was completed within 3 hours of Time 0, they were marked as passed. Blood draw and antibiotic administration done in the correct order (ie, blood draw first) was marked as passed. If all criteria were passed, the overall encounter was marked as having passed the SEP-1 3-hour bundle. If any one of the criteria were failed, however, the encounter was marked as having failed the bundle.

### Other Data Points

Several data points were included in the Slicer Dicer search and downloaded directly from Epic, including sex, race, ethnicity, age at visit, arrival method, disposition, encounter date, and arrival time. Three coders collected the following additional data points from Epic after dividing the encounter list: insurance type (none, Medicaid, Medicare, commercial) and presence of a psychiatric diagnosis at the time of the ED encounter, if any. The presence of psychiatric diagnoses was determined by reviewing the problem list and medical diagnoses noted during the encounter.

Two additional coders blindly recoded data for 63% (n=652) of the encounters. One of the initial coders compared the first and second passes to calculate interrater reliability. Each encounter had 30 data points or chances to agree; therefore, there were 19,560 chances to agree overall. In all, 928 (4.74%) disagreements were identified, indicating that coders agreed 95.26% of the time, for almost perfect interrater reliability.^19^ Furthermore, all 928 disagreements were resolved by checking the medical record to determine which version of the data was correct and including the appropriate set of data in the final database.

### Data Analysis

With the exception of age, all demographic and outcome variables are categorical. Means and standard deviations are reported for age, whereas the remaining demographic variables are reported in terms of frequency and percentage. An independent samples *t* test was used to examine the primary outcome by age, whereas chi-square analyses were conducted to identify relationships between categorical demographic variables, including the presence or absence of a psychiatric diagnosis at the time of the encounter and the outcome. All chi-square tests with a *P*<0.05 were considered significant.

## RESULTS

Of 1,033 encounters examined for adherence to SEP-1 3-hour bundle criteria, 235 did not have a Time 0 event. These encounters were excluded, and the 798 unique patient encounters that did have a Time 0 event were analyzed. Table 1 shows the demographic variables for these patients.

**Table 1.** Demographic Variables

Variable	Overall, n=798	Criteria Met, n=546	Criteria Not Met, n=252
Age, years, mean ± SD	58.70 ± 16.82	59.20 ± 16.28	57.62 ± 17.91
Sex			
Male	421 (52.8)	298 (54.6)	123 (48.8)
Female	377 (47.2)	248 (45.4)	129 (51.2)
Race			
Black	333 (42.1)	212 (39.2)	121 (48.4)
White	434 (54.9)	311 (57.5)	123 (49.2)
American Indian/Alaskan Native	2 (0.3)	2 (0.4)	0 (0.0)
Native Hawaiian/Other Pacific Islander	1 (0.1)	1 (0.2)	0 (0.0)
Asian	5 (0.6)	4 (0.7)	1 (0.4)
Asian Indian	2 (0.3)	1 (0.2)	1 (0.4)
Other	14 (1.8)	10 (1.8)	4 (1.6)
Missing (not included in the percentage calculations)	7 (–)	5 (–)	2 (–)
Ethnicity			
Hispanic/Latinx	16 (2.1)	11 (2.1)	5 (2.0)
Other Hispanic/Latinx	3 (0.4)	1 (0.2)	2 (0.8)
Not Hispanic/Latinx	760 (97.6)	522 (97.8)	238 (97.1)
Missing (not included in the percentage calculations)	19 (–)	12 (–)	7 (–)
Arrival method			
Private vehicle	349 (43.7)	230 (42.1)	119 (47.2)
Ambulance	438 (54.9)	308 (56.4)	130 (51.6)
Other (med flight, ambulatory)	11 (1.4)	8 (1.5)	3 (1.2)
Emergency department disposition			
Admitted or sent to operating room	763 (95.6)	526 (96.3)	237 (94.0)
Transferred	17 (2.1)	7 (1.3)	10 (4.0)
Died	9 (1.1)	7 (1.3)	2 (0.8)
Discharged	7 (0.9)	6 (1.1)	1 (0.4)
Left against medical advice after 3 hours	2 (0.3)	0 (0.0)	2 (0.8)
Insurance			
None	33 (4.1)	22 (4.0)	11 (4.4)
Medicaid	202 (25.3)	131 (24.0)	71 (28.2)
Medicare	437 (54.8)	298 (54.6)	139 (55.2)
Commercial	126 (15.8)	95 (17.4)	31 (12.3)
Presence of a psychiatric diagnosis			
No diagnosis on file during encounter	432 (54.1)	289 (52.9)	143 (56.7)
Diagnosis on file during encounter	366 (45.9)	257 (47.1)	109 (43.3)
Sepsis category			
Severe sepsis	597 (74.8)	390 (71.4)	207 (82.1)
Septic shock	201 (25.2)	156 (28.6)	45 (17.9)

Note: Data are presented as n (%) unless otherwise indicated.

### Group Equivalence

The presence of a psychiatric diagnosis was not related to sex, χ^2^(1)=3.47, *P=*0.062, or race (dichotomized as Black or White), χ^2^(1)=0.80, *P=*0.371. Patients without a psychiatric diagnosis were significantly older (mean age 60.21 ± 16.94 years) than patients with a psychiatric diagnosis (mean age 56.92 ± 16.52 years), *t*(796)=2.76, *P=*0.006.

### SEP-1 3-Hour Bundle Pass/Fail

Overall, bundle criteria were not met in 252 (31.6%) encounters. The presence of a psychiatric diagnosis during the encounter was not significantly related to failure of SEP-1 criteria, χ^2^(1)=1.01, *P=*0.315. All individual psychiatric diagnosis categories were also not significantly related to meeting criteria, with all *P* values ≥0.413.

### Pass/Fail Individual Criteria

Table 2 shows failure rates by individual SEP-1 3-hour bundle criterion. The most frequently failed criterion was the administration of broad-spectrum antibiotics within the 3-hour window. Next was the blood culture draw, followed by the culture being drawn before the antibiotic was administered, and then lactate being drawn. Having a psychiatric diagnosis during the encounter was unrelated to failing any of the criteria.

**Table 2.** SEP-1 3-Hour Bundle Individual Criterion Adherence

	Overall, n=798		No Psychiatric Diagnosis, n=432		Psychiatric Diagnosis, n=366			
Criterion	Failed	Met	Failed	Met	Failed	Met	χ^2^(1)	*P* Value
Lactate	28 (3.5)	770 (96.5)	15 (3.5)	417 (96.5)	13 (3.6)	353 (96.4)	0.004	0.951
Blood culture	117 (14.7)	681 (85.3)	58 (13.4)	374 (86.6)	59 (16.1)	307 (83.9)	1.150	0.284
Antibiotics	166 (20.8)	632 (79.2)	96 (22.2)	336 (77.8)	70 (19.1)	296 (80.9)	1.153	0.283
Culture first	108 (13.5)	690 (86.5)	61 (14.1)	371 (85.9)	47 (12.8)	319 (87.2)	0.277	0.599

Note: Data are presented as n (%).

## DISCUSSION

Sepsis is an illness with an objective, measurable algorithm for its initial assessment and treatment that permits evaluation of the standard of care provided. SEP-1 criteria were met for 68.4% of patients in our study, and our analysis did not find a difference between the care given to patients with or without mental illness. This finding is at odds with previous work showing differences in the care of patients with mental illness in a variety of settings.^20-22^ One explanation for this discrepancy may be that septic patients are quite ill and the impact of potential biases may be overridden by the severity of the illness. Rhee et al found that explicit symptoms of infection were significantly associated with SEP-1 compliance.^23^ We postulate that the presence of evidence-based guidelines had an impact on this lack of bias in the results and could be a model for improving the care of patients in other marginalized groups. Work showing that patients with severe mental illness are at higher risk for mortality when they experience an infection^3,6-9^ adds urgency to exploring whether differential clinical decision-making plays a role. Reinforcing adherence to treatment guidelines and algorithms rather than focusing on interventions to reduce negative attitudes or beliefs (which often do not result in appreciable long-term differences in implicit biases^24^ and have only moderate effects on stigmatizing attitudes^25^) may be one way to help address the issue of differential outcomes and the potential impact of bias on decision-making.

A major limitation of our study is the size of our study population. Studies that have demonstrated correlations between a psychiatric diagnosis and clinical decisions had cohorts between 39,839 and 287,991 patients.^22,26,27^ The methodology of using Slicer Dicer to identify cases has some limitations, and we may not have identified all the potential cases during the time period we were observing. Also, the psychiatric diagnoses and the diagnosis of sepsis could have been inaccurate and/or poorly supported with a deeper dive into the medical records. In addition, correlation or lack of correlation of clinicians’ attitudes toward mental illness with clinical decisions would have provided more insight into the effect of physician attitudes on clinical decision-making.

## CONCLUSION

This study showed no differences in clinical decision-making for people with sepsis and a psychiatric diagnosis of mental illness. The presence of objective guidelines may have lessened the potential role of biases among clinicians toward patients with mental illness. Future work should focus on trying to elucidate the reasons why clinicians often demonstrate different clinical decision-making in patients with mental illness. The use of treatment guidelines has been proposed as a way to lessen these differences and improve outcomes. This matter is not inconsequential given the significantly shortened lifespan for people with serious mental illness. More study and work related to these issues could have a significant impact on the lives of people with serious mental illness.

## Notes

*This article meets the Accreditation Council for Graduate Medical Education and the American Board of Medical Specialties Maintenance of Certification competencies for Patient Care and Medical Knowledge.*
